# Cord blood IgG and the risk of severe *Plasmodium falciparum* malaria in the first year of life

**DOI:** 10.1016/j.ijpara.2016.09.005

**Published:** 2017-02

**Authors:** Linda M. Murungi, Klara Sondén, Dennis Odera, Loureen B. Oduor, Fatuma Guleid, Irene N. Nkumama, Mark Otiende, David T. Kangoye, Greg Fegan, Anna Färnert, Kevin Marsh, Faith H.A. Osier

**Affiliations:** a Kenya Medical Research Institute, Centre for Geographic Medicine Research, Coast, P.O. Box 230-80108, Kilifi, Kenya; b Unit of Infectious Diseases, Department of Medicine, Solna, Karolinska Institutet, SE-171 76 Stockholm, Sweden; c Centre National de Recherche et de Formation sur le Paludisme (CNRFP), 01 BP 2208, Ouagadougou 01, Burkina Faso; d Department of Infectious Diseases, Karolinska University Hospital, Stockholm, Sweden; e African Academy of Sciences, P.O. Box 24916-00502, Nairobi, Kenya; f Nuffield Department of Medicine, Centre for Clinical Vaccinology and Tropical Medicine, University of Oxford, Churchill Hospital, Oxford, United Kingdom

**Keywords:** Antibody, Cord blood, Severe malaria, Merozoite

## Abstract

Young infants are less susceptible to severe episodes of malaria but the targets and mechanisms of protection are not clear. Cord blood antibodies may play an important role in mediating protection but many studies have examined their association with the outcome of infection or non-severe malaria. Here, we investigated whether cord blood IgG to *Plasmodium falciparum* merozoite antigens and antibody-mediated effector functions were associated with reduced odds of developing severe malaria at different time points during the first year of life. We conducted a case-control study of well-defined severe *falciparum* malaria nested within a longitudinal birth cohort of Kenyan children. We measured cord blood total IgG levels against five recombinant merozoite antigens and antibody function in the growth inhibition activity and neutrophil antibody-dependent respiratory burst assays. We also assessed the decay of maternal antibodies during the first 6 months of life. The mean antibody half-life range was 2.51 months (95% confidence interval (CI): 2.19–2.92) to 4.91 months (95% CI: 4.47–6.07). The rate of decline of maternal antibodies was inversely proportional to the starting concentration. The functional assay of antibody-dependent respiratory burst activity predicted significantly reduced odds of developing severe malaria during the first 6 months of life (Odds ratio (OR) 0.07, 95% CI: 0.007–0.74, *P* = 0.007). Identification of the targets of antibodies mediating antibody-dependent respiratory burst activity could contribute to the development of malaria vaccines that protect against severe episodes of malaria in early infancy.

## Highlights

• Severe malaria episodes are rare during the first few months of life.
• The rate of decay of cord blood IgG is inversely proportional to the starting concentration.
• Antibody dependent respiratory burst mediated by cord IgG protects from severe malaria during the first 6 months of infancy.

## Graphical abstract

**Figure:**
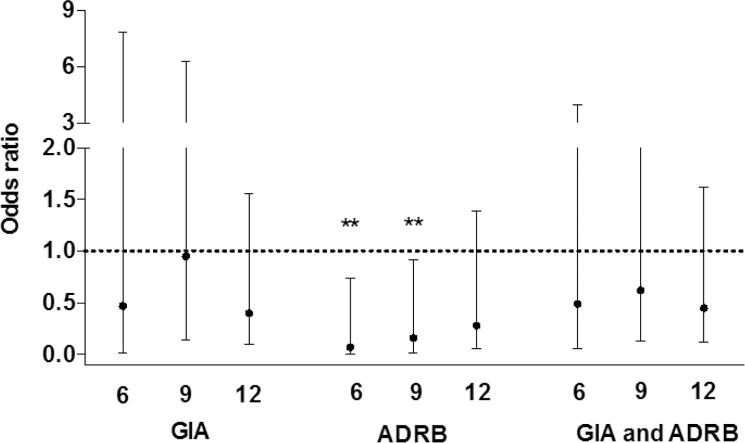

## 1 Introduction

*Plasmodium falciparum* is a leading cause of childhood morbidity and mortality with approximately 214 million cases and 438,000 deaths reported globally in the year 2015 (World Health Organization, 2015). A disproportionate number of the malaria-related deaths occur in sub-Saharan Africa with children under the age of 5 years being at the highest risk of severe and life-threatening malaria. Severe malaria in children manifests in three overlapping clinical syndromes: severe anemia, impaired consciousness and respiratory distress (Marsh et al., 1995). The presentation of these clinical features varies with host age and the level of malaria transmission (Snow et al., 1994, Snow et al., 1997, Roca-Feltrer et al., 2010). In high transmission areas, severe anemia is predominant and affects mainly children aged less than 24 months, while in low-moderate transmission areas cerebral malaria is the main clinical manifestation in older children (O’Meara et al., 2008, Roca-Feltrer et al., 2010), causing high mortality despite appropriate intervention. A significant proportion of those who recover develop long-term neurological and cognitive deficits (Idro et al., 2005).

Young infants in malaria endemic countries are relatively resistant to severe malaria (Snow et al., 1998). Cord blood antibodies are thought to confer protection against clinical episodes of malaria (Edozien et al., 1962) but the evidence is far from clear (Riley et al., 2001, Dobbs and Dent, 2016). Although passively transferred cord blood IgG was shown to reduce parasitemia and clinical symptoms in one study (Edozien et al., 1962), the targets of such antibodies have yet to be identified (Hviid and Staalsoe, 2004). Importantly, although many studies have investigated maternal antibodies in relation to the risk of infection, clinical or febrile malaria, none have focused on severe malaria as the endpoint of interest.

We designed a case-control study of severe malaria nested within a longitudinal birth cohort of infants who were monitored for episodes of well-characterised severe malaria (Lundblom et al., 2013, Murungi et al., 2016). We identified the sub-group of infants for whom a cord blood sample was available. We measured cord blood plasma total IgG levels against five recombinant *P. falciparum* merozoite antigens and its functional activity in the growth inhibition activity (GIA) and antibody-dependent respiratory burst (ADRB) assays (Llewellyn et al., 2015, Murungi et al., 2016). We investigated factors that were likely to have an influence on these antibody measures and assessed the decay of antigen-specific cord blood IgG over the first 6 months of life. Finally we investigated whether antibody levels and function in cord blood were associated with reduced odds of developing severe *falciparum* malaria at different time points during the first year of life when maternal antibodies are likely to persist.

## 2 Materials and methods

### 2.1 Study site and population

The study was conducted in Kilifi County, on the Kenyan coast. The area experiences two seasonal peaks in malaria transmission (May to August and October to November). The study setting and study population are described in detail elsewhere (Lundblom et al., 2013, Murungi et al., 2016). Briefly, following informed consent, infants born to mothers who delivered at Kilifi County Hospital (KCH) or those attending the immunisation clinic during the first month of life were recruited into a birth cohort (Kilifi Birth Cohort, KBC) (*n* = 5,949) set up between 2001 and 2008 to study the risk factors of invasive pneumococcal disease in young children. As the study was primarily set up to study pneumococcal disease, malaria-specific indices such as intermittent preventive treatment during pregnancy (iPTp) and bed net usage were not recorded. The children were followed up quarterly at the Outpatient Department of KCH until 2 years of age. During the quarterly visits, a blood sample was collected and thick and thin blood smears prepared for detection of parasites by microscopy. In the event of an illness outside the scheduled 3-monthly visits, parents were advised to seek care at KCH and the children were treated according to national guidelines. Children who were admitted to hospital were identified using a unique number that linked their clinical, demographic and laboratory information.

#### 2.1.1 Study design

We designed a matched case-control study of well-defined severe malaria cases that included all infants enrolled into the KBC and longitudinally monitored as described in Section 2.1. We included cases admitted to hospital between April 2002 and January 2010. Cases were individually matched to a maximum of three controls by age, date of sample collection and area of residence. Controls were selected from KBC participants who did not present to KCH with severe malaria during the 8-year monitoring period. A total of 61 severe malaria cases were identified and these were individually matched to 161 controls (Lundblom et al., 2013, Murungi et al., 2016). The data presented here are drawn from the subset of these children who were recruited at birth and had a 2 ml venous blood sample taken from the umbilical vein (*n* = 130). Following informed consent, baseline information (age of the mother, number of previous pregnancies, antenatal clinic attendance, gestation period, birth weight and gender of the infant) and a cord blood sample were obtained (Table 1). We also analyzed samples collected at 3 and 6 months of age from the cases and controls to determine the dynamics of decay of maternal antibodies.

#### 2.1.2 Severe malaria case definition

Inclusion criteria for severe malaria cases were admission to hospital between April 2002 and January 2010 with detectable parasites by microscopy and one of the following symptoms: (i) impaired consciousness (Blantyre Coma Score <5), (ii) chest indrawing or deep breathing or (iii) severe anemia (Hb <5 g/dL).

#### 2.1.3 Detection of asymptomatic infections

Detection of malaria parasites in the samples collected every 3 months was performed retrospectively by microscopy and PCR as previously described (Lundblom et al., 2013). Briefly, thick and thin blood films were stained with Giemsa and examined by light microscopy. Parasite densities were determined as the number of parasites per 8,000 white blood cells per μl of blood. The prevalence of submicroscopic infections was determined by PCR amplification of the polymorphic block 3 region of the merozoite surface protein 2 (*msp2*) gene followed by capillary electrophoresis (Liljander et al., 2009).

### 2.2 Recombinant *P. falciparum* merozoite antigens

We measured total IgG titres to a panel of five recombinant merozoite antigens that are currently being assessed in clinical, pre-clinical, animal model and in vitro studies as potential blood-stage malaria vaccine candidates. Reactivity to schizont extract was used as a marker of previous exposure to infection. Full-length apical membrane antigen (AMA)1 (3D7 *P. falciparum* strain) was expressed as a Histidine (His)-tagged protein in *Pichia pastoris* (Dutta et al., 2002), MSP-2 (Dd2 *P. falciparum* strain) was expressed as a glutathione *S-*transferase (GST)-fusion protein in *Escherichia coli* (Taylor et al., 1995) and MSP-3 (3D7) as a maltose binding protein (MBP)-fusion protein also in *E. coli* (Polley et al., 2007). The C-terminal 19 kDa fragment of MSP-1 (MSP-1_19_) (Wellcome) and a fragment of *P. falciparum* reticulocyte-binding homolog 2 (*Pf*Rh2 (3D7)) were expressed as GST- (Burghaus and Holder, 1994) and His-tagged fusion proteins (Reiling et al., 2010), respectively, in *E. coli.* A *P. falciparum* schizont lysate based on the A4 parasite line was prepared by sonicating mature schizont stages (Ndungu et al., 2002).

### 2.3 ELISA

Total IgG responses against the *P. falciparum* merozoite antigens described in Section 2.2 were simultaneously measured by multiplex ELISA as described previously (Rono et al., 2013). We also measured IgG to parasite schizont lysate (3D7) using a standard ELISA protocol (Murungi et al., 2016). Eleven serial dilutions of a purified IgG preparation obtained from Malawian adults (Taylor et al., 1992) were included for every antigen tested to obtain a standard dilution curve that allowed the conversion of median fluorescence intensity (MFI) readings to arbitrary antibody concentrations. A pool of plasma obtained from Kilifi adults was included in a single well on each plate as a positive control to allow for standardisation of day-to-day and plate-to-plate variation. Twenty plasma samples obtained from UK adults who had not been exposed to malaria were also included as negative controls for each antigen tested. Seropositivity for antibody titres was defined as ELISA O.D. value above the mean + 3 S.D. of the 20 malaria non-exposed UK plasma samples. All samples were assayed in duplicate and for those that had a coefficient of variation (CV) greater than 20%, the assays were repeated.

### 2.4 Assays of antibody function

The assays of GIA and ADRB activity were performed as previously described (Murungi et al., 2016) using cord blood plasma samples. The GIA assay has been used to assess the vaccine efficacy of blood-stage vaccine candidates both in animal models and clinical studies (Duncan et al., 2012). The assay has also been associated with protection from clinical malaria in some studies (Crompton et al., 2010, Rono et al., 2012) but this has not been a consistent finding. The ADRB assay has also been shown to correlate with protection against clinical episodes of malaria in field studies (Joos et al., 2010).

#### 2.4.1 Assay of GIA

Cord plasma were dialyzed in 1 X PBS using 20 kDa MWCO mini dialysis units and incubated at 56 °C for 30 min to inactivate complement proteins. Highly synchronous trophozoite stage parasites from the 3D7 *P. falciparum* strain (0.3–0.5% parasitemia, 1% hematocrit) were added to individual wells, followed by the dialyzed plasma at a ratio of 1:10. The plates were incubated in a humidified chamber containing 5% CO_2_, 5% 0_2_ and 90% N_2_ for 80 h. Ten microliters of culture medium were added to each well after the first growth cycle. A positive and negative control well containing 10 mg/ml of purified Malaria Immune Globulin (MIG) and a pool of plasma from UK adults, respectively, were included. After two cycles, the parasites were stained with 10 μg/ml of ethidium bromide for 30 min, washed with 1 X PBS and acquired on an FC500 (Beckman Coulter, USA) flow cytometer.

#### 2.4.2 Parasite culture and isolation of parasitophorous vacuole enclosed membrane structures (PEMS)

*Plasmodium falciparum* 3D7 parasite cultures were maintained at <10% parasitemia and 2% hematocrit. Highly synchronous mature trophozoite stages were enriched by magnetic separation and allowed to mature into early schizont stages. Thereafter, a protease inhibitor (trans-epoxysuccinyl-L-leucylamido (4-guanidino) butane (E-64)) was added to allow development into late schizonts without rupture. The schizonts were pelleted, resuspended in 1 X PBS and stored at −20 °C.

#### 2.4.3 Isolation of human polymorphonuclear cells (PMNs)

Whole blood samples from healthy donors were collected into heparin tubes, layered onto Ficoll-Histopaque 1077 (Sigma, Germany) and centrifuged at 600*g* for 15 min at room temperature (RT). The pellet containing PMNs and red blood cells (RBCs) was resuspended in 3% dextran solution and incubated for 30 min at RT in the dark. Thereafter, the supernatant was carefully removed and centrifuged at 500*g* for 7 min. Residual RBCs were lysed using ice cold 0.2% NaCl followed by 1.6% NaCl. PMNs were washed in PMN buffer (Hank’s Balanced Salt Solution (HBSS) supplemented with 0.1% BSA and 1% D-(+)-glucose) and resuspended in PMN buffer at a concentration of 1x10^7^ PMNs/ml.

#### 2.4.4 ADRB assay

The ADRB assay was performed as previously described (Llewellyn et al., 2015, Murungi et al., 2016). Briefly, PEMS were thawed and coated overnight onto individual wells of Nunc opaque MaxiSorp 96-well plates (Thermo Scientific, USA) at 18.5 × 10^5^/ml. Following three washes with 1 X PBS, the plates were blocked and incubated with plasma (1:50) for 1 h at 37 °C. The plates were washed and 50 μl of PMNs (1 × 10^7^ PMNs/ml) added, followed by 50 μl of isoluminol (0.04 mg/ml). Chemiluminescence was measured for 1 s every 2 min over an hour. Control wells containing a pool of UK adult plasma and a pool of plasma from Kilifi adults were included in each plate. Readings were expressed relative to those obtained from the pool of plasma from Kilifi adults.

### 2.5 Statistical analysis

All analyses were performed using Stata 13.0 (StataCorp, Texas, USA) and GraphPad prism Version 6 (GraphPad Software, Inc). A linear regression model that included an indicator of the case-control status was used to determine the influence of maternal age, parity, birth weight, year of birth, season of birth and gestation period on the levels of specific antibodies and antibody function. The Benjamini-Hochberg method was used to adjust for the false discovery rate. The rate of antibody decay was determined using a longitudinal mixed-effects model (Amanna et al., 2007, Fowkes et al., 2012). The model accounts for repeated measurements from an individual and was fitted to the log_10_-transformed values of the antibody titres collected at ages 0, 3 and 6 months. To assess the natural decay of maternal IgG in the absence of boosting due to infection, we excluded children that had antibody titres boosted at 3 months of age compared with baseline titres as well as those with boosted titres at 6 months of age compared with titres at 3 months of age. Half-life estimates were calculated using the equation:

$$T_{1/2}=\ln2/m$$

where *T*_1/2_ is the estimated half-life and *m* is the slope component of the mixed-effects model (Amanna et al., 2007, Kinyanjui et al., 2007, Ochola et al., 2009, Fowkes et al., 2012). A conditional logistic regression model was used to investigate the association between antibody levels and function with the odds of developing a severe malaria episode at different time points over the first year of life. Antibodies were fitted as categorical variables in the model based on a cut-off for seropositivity (defined as an ELISA O.D. value above the mean + 3 S.D. of 20 European plasma samples). This cut-off was also used to define the seroprevalence of merozoite-specific antibodies. The Wald test was used to estimate the statistical significance of categorical covariates. Seropositivity for ADRB was defined as a cut-off above the mean + 3 S.D. of 20 European plasma samples whereas the GIA cut-off for positivity was defined as being above the median GIA level of the cord plasma samples assayed (Rono et al., 2012, Murungi et al., 2016). *P* < 0.05 was considered statistically significant.

## 3 Results

### 3.1 Severe malaria episodes

**Fig. 1:**
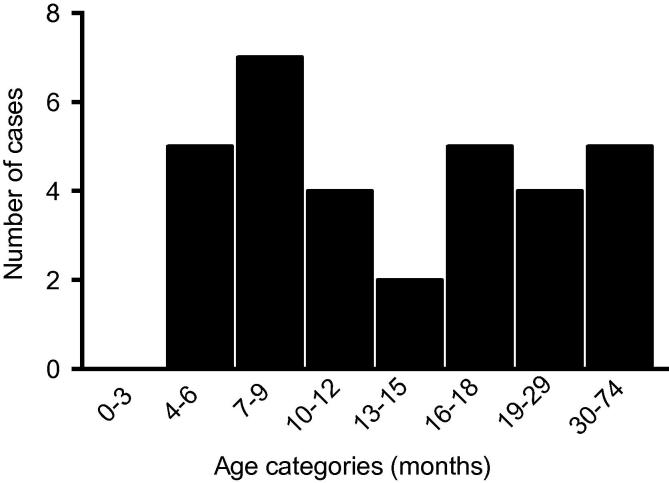
The number of severe malaria cases recorded at different time points from birth up to 74 months of age (*n* = 32) during the study period.

Of the 222 children recruited to the matched case-control study of severe malaria described previously (Lundblom et al., 2013, Murungi et al., 2016), 130/222 (58.5%) were born at KCH and had a 2 ml cord blood sample drawn at birth. Of these, 32 developed severe malaria; 12 (37%), seven (21%) and six (18%) of whom presented to hospital with respiratory distress, impaired consciousness (BCS <5) and severe malaria anemia (Hb <5 g/dL), respectively. Six infants (18%) presented with two overlapping severe malaria syndromes and one (3%) with all three syndromes. Of the 32 cases, five (15.6%) occurred during the first 6 months of life, 12 (37.5%) within 9 months and 16 (50%) before 12 months. The remaining 16 cases (50%) occurred beyond the age of 1 year. No cases occurred in the first 4 months of life (Fig. 1). The median age of admission with severe malaria was 12.5 months (range 5.6–74.0). The remaining 98/130 (75%) children made up the controls, 20 of whom had a history of admission to hospital with gastroenteritis and lower respiratory tract infections. None of the controls was admitted with non-severe malaria.

Twenty-seven out of 130 (20.0%) acquired asymptomatic *P. falciparum* infections as measured by PCR or microscopy in the quarterly samples collected up to 2 years of age. Of these, 11/32 (34%) were identified among the children who subsequently developed severe malaria and 16/98 (16%) among the controls.

### 3.2 Prevalence of antibodies and functional indices of immunity

**Fig. 2:**
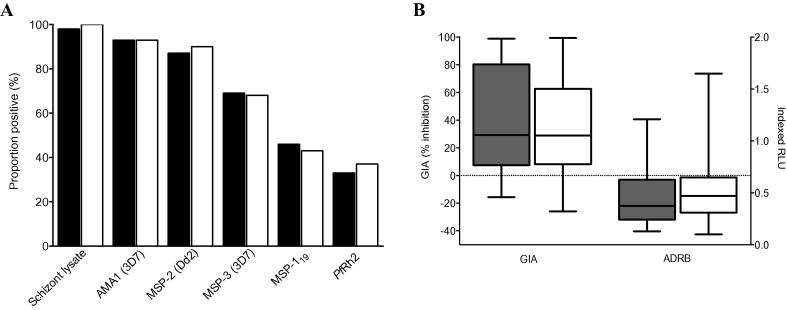
Antibody seroprevalence, growth inhibition activity (GIA) and neutrophil antibody-dependent respiratory burst (ADRB) levels among the cases and controls. (A) The seroprevalence to different merozoite antigens between the cases (black bars) and controls (white bars) are shown. (B) Box-and-whisker plots showing the levels of GIA and ADRB among the cases (grey bars) and controls (white bars). The horizontal lines represents the medians and interquartile ranges; whiskers show the maximum and minimum values. Negative GIA values indicate enhanced growth of parasites in the presence of test sera compared with the untreated culture. Relative light unit (RLU) values greater than 1 indicate higher ADRB activity induced by serum samples compared with the semi-immune serum pool.

There was no significant difference in cord blood seroprevalence (Fig. 2A) or levels of antibodies (Supplementary Fig. S1) between the cases of severe malaria (*n* = 32) and controls (*n* = 98) for all antigens. Among the cases, the seroprevalence against schizont lysate, AMA1(3D7), MSP-2(Dd2), MSP-3(3D7), MSP-1_19_ and *Pf*Rh2 was 98%, 93%, 87% 69%, 46% and 33%, respectively, compared with 100%, 93%, 90%, 68%, 43% and 37% among the controls (Fig. 2A). The median GIA level was 28.9% (range −25.9 to 99.4) with no statistically significant difference between the cases and controls (29.2% and 28.9%, respectively) (Fig. 2B). Similarly, the median ADRB level was 0.4 indexed relative light units (RLU) (range 0.1–1.65) and the median levels were comparable among the cases and controls (0.3 and 0.4, respectively) (Fig. 2B). We previously published threshold concentrations of antibodies to specific antigens that appeared to be necessary for protection against clinical episodes of malaria (Murungi et al., 2013, Rono et al., 2013). The prevalence of antibodies at threshold concentrations in cord blood was low and importantly did not differ between severe malaria cases and controls; AMA1, 6.2% versus 6.1%; MSP-2, 21.8% versus 18.3% and MSP-3, 18.7% versus 17.7%, respectively.

### 3.3 Factors that influence the level and function of antibodies measured in cord blood plasma

We used a linear regression model to determine which factors were positively or negatively correlated with increasing antibody levels and function in cord blood plasma (Table 2). Maternal age was positively associated with higher GIA and ADRB levels (regression coefficient 0.94 (0.07, 1.81), *P* = 0.03 and 0.01 (0.003, 0.01), *P* = 0.004, respectively) (Table 2) and (Supplementary Fig. S2) but not with antibodies to individual antigens (Table 2). However, this result remained significant for ADRB levels only after adjustment for multiple comparisons (*P* = 0.02). Surprisingly, we observed an increase in GIA and ADRB levels over the duration of recruitment (2002–2006) (Supplementary Fig. S3), a period marked by continuous decline in malaria transmission intensity (Okiro et al., 2007, O’Meara et al., 2008). Other factors such birth weight, parity, gestation period, season and gender of the child did not significantly influence the levels of specific antibodies measured in cord blood or their functional activity (Table 2).

### 3.4 Dynamics of the decay of antibody titres

The decay rate of antibodies to specific antigens was determined using a longitudinal mixed-effects model (Amanna et al., 2007, Fowkes et al., 2012). A regression line was fitted through log_10_ transformed antibody titres for both cases and controls aged less than 6 months. All the index samples were collected prior to the severe malaria episode. To assess the decay of antibodies in the absence of boosting of *P. falciparum* infection, we excluded results from five cases and five controls who had detectable parasites at either month 3 or 6. In addition, all samples from individuals whose antibody titres at 3 months of age were higher than cord titres were excluded and for those whose titres at 6 months were higher than month 3, only the result for the 6-monthly sample was excluded. A total of 56 (43%) samples (12 cases and 44 controls) were excluded in the analysis. The mean half-life range was 2.51 months (95% confidence interval (CI): 2.19–2.92) to 4.91 months (95% CI: 4.47–6.07) (Table 3). There was no significant difference in the mean decay rate of antibodies against AMA1 (3D7), MSP-2 (Dd2) and MSP-3 (3D7). However, anti-MSP-1_19_ and *Pf*Rh2 antibodies had a significantly longer mean half-life compared with other antigens tested (mean half-life 4.27 months (3.85–5.02) and 4.91 months (4.47–6.07, respectively) (Table 3). The rate of decay of antibodies did not differ between infants who subsequently became cases and those that were controls although the numbers in each group were limited and precluded the ability to detect any significant differences (Supplementary Table S1).

**Fig. 3:**
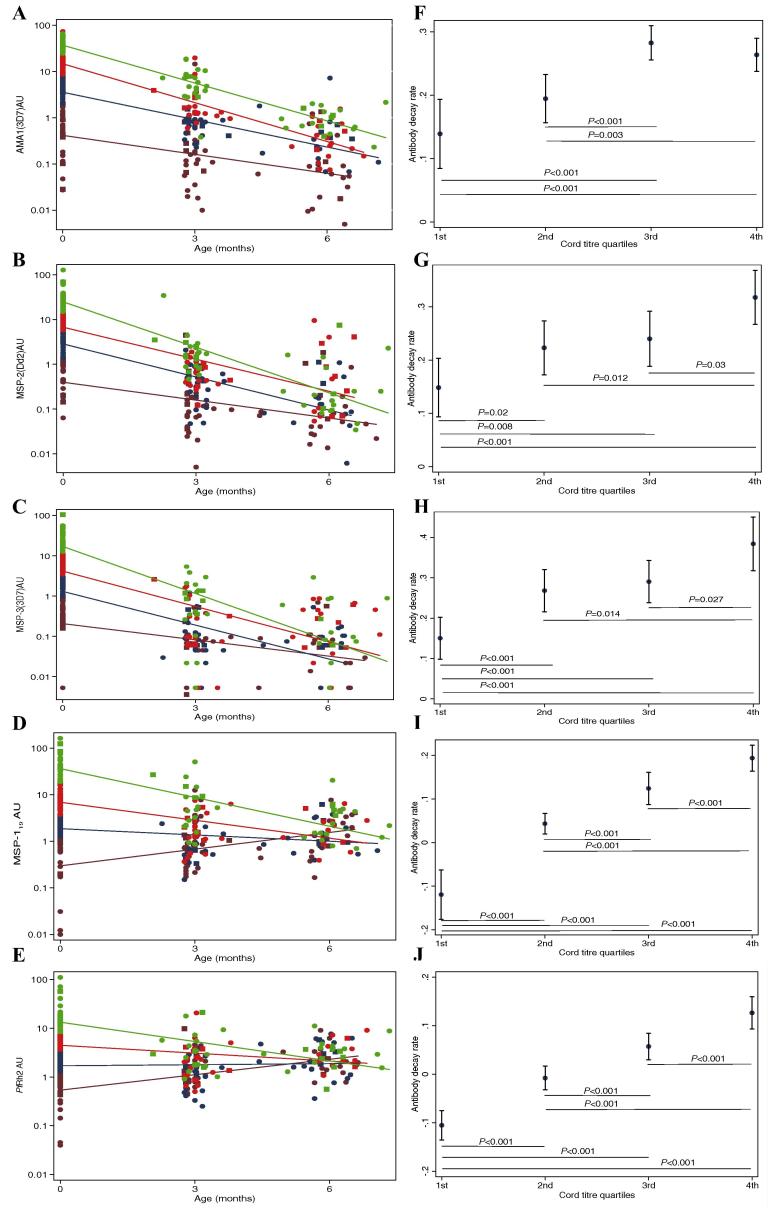
Decay of cord blood IgG relative to the initial concentration. Scatter plots of antibody titres to (A) apical membrane antigen 1 (AMA1) (3D7 *Plasmodium falciparum* strain), (B) merozoite surface protein 2 (MSP-2) (Dd2 *P. falciparum* strain), (C) MSP-3 (3D7) (D) MSP-1_19_ and (E) *P. falciparum* reticulocyte-binding homolog 2 (*Pf*Rh2), showing the decay rates relative to the initial (cord blood) titres. Closed squares and circles represent the cases and controls, respectively. Antibody titres in cord blood were divided into quartiles and regression lines fitted for each quartile as shown in maroon (1st quartile), blue (2nd quartile), red (3rd quartile) and green (4th quartile) using data collected during 6 months of follow-up from birth. The decay rates and 95% confidence intervals (CIs) of cord blood titres (y-axis) according to the different quartile levels (x-axis) are shown for the antigens tested (F) AMA1 (3D7), (G) MSP-2 (Dd2), (H) MSP-3 (3D7), (I) MSP-1_19_ and (J) *Pf*Rh2. *P* values <0.05 indicate differences between regression coefficients (decay rates) that were statistically significant.

Next, we tested the rates of decay of antibodies according to the quartile distribution of cord titres. The titres were divided into quartiles and the decay rate of antibodies within each quartile assessed as shown in Fig. 3. Titres in the highest quartile (4th quartile) decayed most rapidly, followed by those in the 3rd, 2nd and 1st quartiles, in that order (Fig. 3) for all antigens tested with the exception of AMA1 (Fig. 3A). For example, the decay rate for MSP-2 (Dd2) antibodies was 0.14, 0.22, 0.23 and 0.31 log_10_ AU reduction per month in the 1st, 2nd, 3rd and 4th cord quartiles, respectively (Fig. 3B). The average rate of decay and 95% CI estimates across the different quartiles for all antigens tested are shown in Fig. 3F–J.

### 3.5 Relationship between antibodies and odds of developing severe *P. falciparum* malaria

**Fig. 4:**
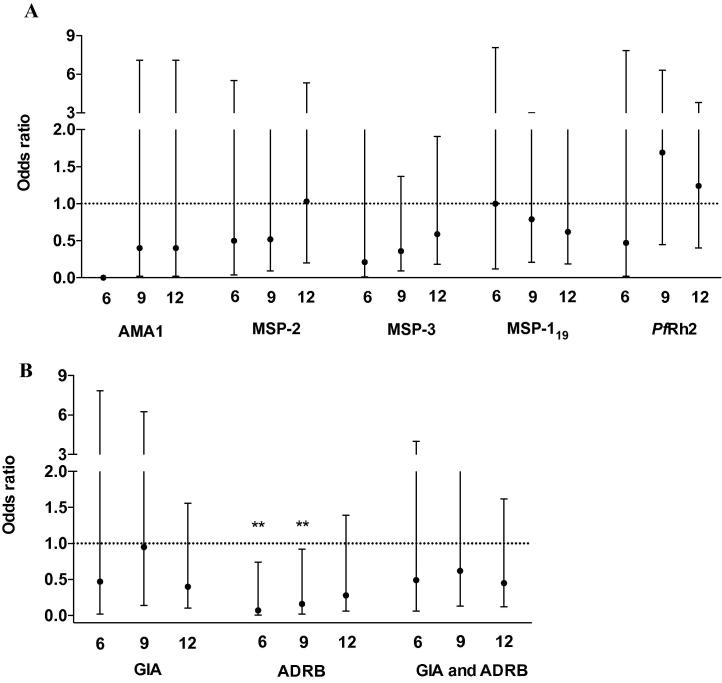
Relationship between antibody titres and function with risk of severe malaria. The association between (A) antibody seropositivity and (B) antibody-mediated function with the odds of developing severe malaria from birth up to 6 months, 9 months and 12 months of age. The plots show the odds ratio (OR) and 95% Confidence Interval obtained from conditional logistic regression models. Estimates marked with asterisks are statistically significant at *P* < 0.05. ND, not determined. The OR estimates could not be determined from the model. AMA, apical membrane antigen; MSP, merozoite surface protein; *Pf*Rh, *P. falciparum* reticulocyte-binding homolog 2; GIA, growth inhibition activity; ADRB, neutrophil antibody-dependent respiratory burst.

We evaluated the relationship between cord antibody titres and their functional activity (GIA and ADRB) with the odds of developing severe malaria at different time points after birth (6, 9 and 12 months of age) (Fig. 4). Although the median age of admission with severe malaria was 12.5 months (range 5.6–74.0), we excluded the cases of severe malaria that occurred beyond 12 months (*n* = 16), as it was highly unlikely that cord blood antibodies were responsible for protection. Thus, we analysed samples from a total of 16 severe cases admitted to hospital during the first 12 months of life. These were individually matched to four controls per case based on date of birth. Three cases were born a few days apart and were therefore matched to a similar set of four controls.

Seropositivity for antibody titres and ADRB were defined as a cut-off above the mean + 3 S.D. of 20 European plasma samples whereas the GIA cut-off for positivity was defined as being above the median GIA level of the cord plasma samples (Rono et al., 2012, Murungi et al., 2016). Of the 130 children who had a cord plasma sample, 26/32 (81%) and 16/32 (50%) severe malaria cases were seropositive for ADRB and GIA, respectively. Of the 16 severe malaria cases admitted to hospital during the first 12 months of life, 2/5 (40%), 8/12 (66%) and 12/16 (75%) were seropositive for ADRB during the first 6, 9 and 12 months of life, respectively, compared with 18/20 (90%), 43/47 (91%) and 50/55 (90%) of controls. On the other hand, 3/5 (60%), 8/12 (66%) and 9/16 (56%) of cases and 12/18 (66%), 29/44 (65%) and 36/59 (70%) of controls had antibodies above the median GIA levels during the first 6, 9 and 12 months of life, respectively.

There was a positive and significant correlation between cord blood antibodies to all the antigens tested in both the severe malaria cases and controls with the exception of antibodies against MSP-2 and *Pf*Rh2 (*rho* = 0.13, *P* = 0.19) and MSP-3 and *Pf*Rh2 (*rho* = 0.16, *P* = 0.10) among the controls but not cases (Supplementary Fig. S4). Assays of GIA and ADRB activity were significantly correlated among the controls (*rho* = 0.34, *P* = 0.0006) but not the cases (*rho* = −0.0009, *P* = 0.99) (Supplementary Fig. S4). There was only limited co-linearity between the different indices of immunity that we measured (VIFs <2; mean VIF = 1.77 and 1.70 in the cases and controls, respectively). We observed a significant increase in ADRB levels (Kruskal–Wallis test *P* < 0.009) but not GIA activity (Kruskal–Wallis test *P* = 0.058) with increase in the breadth of merozoite-specific cord blood antibodies (seropositivity against the number of antigens tested) (Supplementary Fig. S5). Antibody titres to all antigens tested were not associated with protection against severe malaria at the different time points after birth (Fig. 4A). Children who were positive for ADRB had significantly reduced odds of developing severe malaria during the first 6 months of life (OR 0.07 (95% CI: 0.007–0.74) *P* = 0.007) and this association remained strong and significant until 9 months of age (OR 0.16 (95% CI: 0.02–0.92, *P* = 0.041) but was lost thereafter (Fig. 4B). In contrast, GIA was not associated with protection at any time point (Fig. 4B). We have previously demonstrated that children who had antibodies that were capable of inhibiting growth of parasites and mediating release of reactive oxygen species by neutrophils had significantly reduced odds of developing severe malaria during the first year of life (Murungi et al., 2016). Here, a combination of the two functional assays was not associated with protection against severe malaria (Fig. 4B).

## 4 Discussion

We designed a case control study of severe malaria nested within a longitudinally monitored birth cohort. This design provided a unique opportunity to determine whether antibodies present in cord blood were associated with a reduced risk of severe malaria during infancy. We found that cord blood IgG in the assay of ADRB activity was significantly associated with lower odds of developing severe malaria. This association was only observed for severe malaria cases occurring within 9 months of birth and fits expectations of the half-life of passively transferred maternal antibodies (White et al., 2014). Cord blood IgG against merozoite antigens tested was not associated with protection and the half-life ranged from between 2.5 to 4.9 months for the antigens tested. Interestingly, antibody decay rates were inversely proportional to the initial titres present in cord blood.

The ADRB assay has been assessed in a limited number of studies (Joos et al., 2010, Kapelski et al., 2014, Llewellyn et al., 2015) and has been shown to correlate with protection against clinical episodes of malaria in some of these investigations (Joos et al., 2010). No studies have evaluated the effect of cord blood antibodies capable of inducing ADRB with subsequent risk of malaria in early infancy and we propose that our study is unique in this respect. The strong correlation between ADRB levels and breadth of responses to merozoite antigens suggest that multiple targets mediate the overall ADRB activity. On the other hand, the weak correlation between GIA and ADRB highlight the distinct mechanisms of actions measured by these assays and suggest that the merozoite targets may be different or antibodies to the targets that mediate these mechanisms were absent. GIA was not associated with protection against severe malaria.

Our study differs from previously published reports that investigated the protective role of maternal antibodies against the outcomes of infection and/or clinical or febrile malaria (reviewed in Riley et al., 2001, Dobbs and Dent, 2016). None of the previous studies focused on severe malaria as an endpoint. Furthermore, the duration of observation in previous analyses has varied ranging from between 5 months (Riley et al., 2000) to 2 years (Kangoye et al., 2014). Our present analysis supports the view that maternal antibodies against merozoite antigens are unlikely to persist at high titres beyond 5 months (Kangoye et al., 2016, White et al., 2014) and that this relatively short period during which antibodies are actually available may account for the inconsistency in findings from studies investigating the protective role of maternal antibodies (reviewed in Riley et al., 2001, Dobbs and Dent, 2016). Importantly, whether the end-point of such studies is severe or uncomplicated malaria, clinical episodes as a whole are relatively rare in children under 6 months of age despite the fact that they frequently harbor low-level asymptomatic infections (Wagner et al., 1998, D’Alessandro et al., 2012). Thus whilst antibodies may play a protective role, the lack of sufficient cases during this period limits the analysis and will require very large studies.

A key and interesting finding in our study was that antibody decay rates are inversely proportional to the initial titres present in cord blood, a finding that is in contrast to a study by Riley et al. (2000) showing that cord blood antibody titres in Ghanaian infants persisted for a long duration in infants who had antibody levels above the median O.D. level at birth compared with those below the median. However, results similar to ours have been reported from studies of viral infections that demonstrated a rapid postnatal decline of maternally transferred antibodies against rubella (Cloonan et al., 1970), parainfluenza type 3 and influenza A2 (Cloonan et al., 1971) in children with high titres at birth compared with infants with low initial titres. IgG sub-class antibodies against merozoite antigens have also been reported to decay faster than their expected theoretical half-life in older children (Kinyanjui et al., 2007).

We speculate that the rate of decay of maternal antibodies may have been influenced by the presence of sub-patent infections. Moderate to high maternal antibody titres may have a masking effect on infections in infants and this could contribute to the rapid decay of circulating antibodies. On the other hand, infections in the presence of low titre maternal antibodies could result in seroconversion and persistence of antibody titres (Riley et al., 2001). Detection of asymptomatic infections in our study was not comprehensive as sampling was undertaken only once every 3 months.

The distribution of different IgG subtypes could also affect the rate of antibody decline. For instance, IgG3 is more rapidly degraded than other isotypes (Spiegelberg, 1974). In other infections, factors such as nutritional status of the infant, breastfeeding, environmental factors and the presence of other concurrent infections during infancy have not been shown to influence the kinetics of antibody decay (Caceres et al., 2000) but their effect on malaria-specific maternal antibodies is not known.

We found that anti-merozoite antibodies in cord blood were not associated with parity, maternal age, birth weight or gestational age. In contrast, other studies demonstrated a positive relationship between parity and placental malaria infection (Ricke et al., 2000, Mayor et al., 2011) with increased levels of total IgG to var2CSA and merozoite antigens. Multiple factors are thought to influence the transplacental transfer of antibodies and may account for this disparity. These include maternal factors such as differences in maternal titres which vary depending on the antigen, IgG subclass distribution, nature of antigen tested, timing of exposure to antigens during pregnancy and the duration of this exposure (Palmeira et al., 2012). Varying analytical approaches are also applied to the study of maternal antibodies; some studies use cord blood antibody levels as a proxy measure of the efficacy of transplacental antibody transfer while others consider the ratio of antibodies in the mother to infant (Palmeira et al., 2012). We did not have data on placental malaria, HIV infection, IPTp or use of insecticide-treated bed nets, all of which may have an impact on antibody levels.

In summary, we found that cord blood IgG activity in the ADRB assay was strongly associated with lower odds of developing severe malaria in the first 9 months of life. Although larger studies are needed, these data suggest that ADRB could be useful for the identification of targets of protective antibodies that could be translated to the clinic as candidate vaccines for infants and young children who are most susceptible to death due to severe malaria.

## Figures and Tables

**Table 1:** Baseline characteristics of the mothers and their infants at enrolment in the study.

	Cases (*n* = 32)	Controls (*n* = 98)	*P* value^a^
Median maternal age in years (range)^b^	27.8 (14.9–44.0)	25.4 (14.7–48.0)	0.26
Median no. of previous pregnancies (range)^c^	5 (0–8)	4 (0–13)	0.25
Gender female, ***n***/Total (%)	15/32 (46.8%)	47/98 (47.9%)	0.91
Median gestation weeks^d^ (range)^e^	38 (23–42)	38 (28–50)	0.82
Median birth weight in kilograms (range)^f^	2.7 (1.8–4.3)	2.8 (1.6–3.8)	0.68
Antenatal clinic attendance, *n*/Total recorded (%)	12/13 (92.3%)	43/45 (95.5%)	0.64
Year of birth, *n* (%)			
2002	15 (46.8%)	40 (40.8%)	0.56
2003	9 (28.1%)	21 (21.4%)	
2004	6 (18.7%)	31 (31.6%)	
2005	2 (6.2%)	4 (4.0%)	
2006	0 (0%)	2 (2.0%)	
Season of birth			
Dry season^g^	20 (62.5%)	58 (59.1%)	0.74
Rainy season^h^	12 (37.5%)	40 (40.8%)	

a The Mann–Whitney *U* and chi-square tests were applied for comparisons of continuous variables and proportions, respectively, among the cases and controls.

b Data were available from mothers of 32 (100%) severe malaria cases and 96 (97%) controls.

c Data were recorded from mothers of 22 (68%) cases and 74 (75%) controls.

d Gestation age was assessed based on the date of last menstrual period (LMP) or symphyseal-fundal height of the uterus, converted to weeks using dating charts.

e Data were recorded from mothers of 13 (40%) cases and 45 (45%) controls.

f Data were available for 16 (50%) children who developed severe malaria and 50 (51%) controls.

g The dry season occurs in January–March.

h The rainy season occurs in May–August and October–November.

**Table 2:** Factors that influence the levels and function of merozoite-specific antibodies in cord blood plasma.

Predictor (No. cases, No. of controls)	Anti-AMA	*P* *P^∗^*	Anti-MSP-2	*P* *P^∗^*	Anti-MSP-3	*P* *P^∗^*	Anti-MSP-1_19_	*P* *P^∗^*	Anti-Rh2	*P* *P^∗^*	GIA	*P* *P^∗^*	ADRB	*P* *P^∗^*
Maternal factors														
Age (32, 96)	−0.01 (−0.03, 0.007)	0.23 0.33	0.008 (−0.007, 0.02)	0.31 0.40	0.01 (−0.001, 0.03)	0.05 0.11	0.006 (−0.01, 0.02)	0.60 0.98	0.0006 (−0.01, 0.01)	0.93 0.61	0.94 (0.07, 1.81)	**0.03** 0.10	0.01 (0.003, 0.01)	**0.004** **0.02**
Gravidity Primigravid (2, 9) Multigravid (20, 65)	−0.27 (−0.75, 0.20)	0.25 0.56	0.19 (−0.22, 0.62)	0.35 0.56	−0.06 (−0.55, 0.41)	0.78 0.98	−0.01 (−0.59, 0.55)	0.95 0.56	−0.25 (−0.64, 0.14)	0.21 0.98	0.72 (−21.15, 22.59)	0.94 0.98	0.07 (−0.09, 0.24)	0.36 0.56
Gestation (weeks) Full Term (10, 35) Preterm (3, 10)	0.16 (−0.23, 0.57)	0.40 0.56	0.26 (−0.13, 0.65)	0.18 0.33	0.31 (−0.14, 0.77)	0.17 0.33	0.18 (−0.41, 0.79)	0.53 0.70	0.07 (−0.31, 0.46)	0.71 0.61	15.01 (−6.56, 36.59)	0.16 0.33	0.15 (−0.003, 0.31)	0.05 0.33
Infant factors														
Gender Male (17, 51) Female (15, 47)	0.13 (−0.13, 0.41)	0.31 0.71	0.02 (−0.21, 0.26)	0.83 0.89	−0.04 (−0.30, 0.21)	0.72 0.83	0.13 (−0.18, 0.44)	0.41 0.71	−0.09 (−0.30, 0.10)	0.34 0.71	6.25 (−6.09, 18.59)	0.31 0.71	−0.03 (−0.13, 0.06)	0.54 0.77
Birth weight (16. 50)	−0.09 (−0.40, 0.20)	0.51 0.89	0.01 (−0.26, 0.30)	0.90 0.90	−0.06, −0.37, 0.25)	0.70 0.90	−0.04 (−0.47, 0.37)	0.82 0.89	0.09 (−0.18, 0.38)	0.48 0.90	−10.81 (−25.63, 4.00)	0.14 0.89	−0.01 (−0.13,0.09)	0.75 0.89
Environmental factors														
Season Dry season (20, 58) Wet season (12, 40)	−0.09 (−0.36, 0.18)	0.52 0.89	0.03 (−0.20, 0.28)	0.76 0.89	−0.04 (−0.30, 0.21)	0.74 0.89	−0.40 (−0.72, −0.08)	**0.013** 0.07	−0.06 (−0.27, 0.14)	0.53 0.89	0.25 (−12.42, 12.92)	0.96 0.97	0.01 (−0.08,0.11)	0.75 0.89
Year of Birth 2002 (15, 40) 2003 (9, 21) 2004 (6, 31) 2005 & 2006 (2, 6)	−0.10 (−0.45, 0.25) −0.12 (−0.45, 0.21) −0.10 (−0.69, 0.48)	0.88 0.91	0.05 (−0.25, 0.36) −0.14 (−0.43, 0.14) −0.29 (−0.80, 0.22)	0.45 0.87	0.01 (−0.31, 0.34) −0.03 (−0.35, 0.27) −0.41 (−0.96, 0.14)	0.51 0.87	0.15 (−0.25, 0.56) 0.05 (−0.33, 0.44) −0.13 (−0.82, 0.55)	0.83 0.91	−0.04 (−0.31, 0.23) −0.01 (−0.27, 0.23) 0.12 (−0.32, 0.58)	0.91 0.91	**23.35 (8.65, 38.06)** **22.36 (8.31, 36.40)** −22.34 (−46.53, 1.85)	**0.0001** **0.0007**	**0.13 (0.01, 0.26)** **0.20 (0.08, 0.32)** 0.06 (−0.14,0.27)	**0.006** **0.01**

The values indicated in the table are coefficients (95% confidence intervals) and *P* values of the change in relative antibody concentrations or function per unit change in continuous covariate or compared with the reference group, for categorical covariates. Coefficients less than, greater than or equal to zero indicate a decrease, increase or no overall change in antibody concentrations per unit increase in the explanatory variable, respectively. *P* < 0.05 was considered statistically significant. *P^∗^* are adjusted *P* values after correction for multiple testing. Bold indicates *P* values that are statistically significant.

AMA, apical membrane antigen; MSP, merozoite surface protein; *Pf*Rh, *Plasmodium falciparum* reticulocyte-binding homolog; GIA, growth inhibition activity; ADRB, neutrophil antibody-dependent respiratory burst.

**Table 3:** The overall rate of decay and antibody half-lives of maternally transferred antibodies against specific merozoite antigens.

Antigen	Rate of decay^a^ (95% CI)	Antibody half-life in months (95% CI)
AMA1 (3D7)	−0.222 (−0.243, −0.201)	3.121 (2.851–3.447)
MSP-2 (Dd2)	−0.243 (−0.276, −0.210)	2.851 (2.510–3.296)
MSP-3 (3D7)	−0.276 (−0.316, −0.237)	2.510 (2.193–2.924)
MSP-1_19_	−0.162 (−0.180, −0.138)	4.277 (3.850–5.021)
*Pf*Rh2	−0.141 (−0.155, −0.114)	4.914 (4.470–6.078)

CI, Confidence interval; AMA, apical membrane antigen; MSP, merozoite surface protein; *Pf*Rh, *Plasmodium falciparum* reticulocyte-binding homolog; AU, arbitrary units.

a Decay rate (log10 AU/month).
