# Supplementary material for: Cord blood IgG and the risk of severe Plasmodium falciparum malaria in the first year of life
Source: Int J Parasitol. 2017 Feb;47(2-3):153–62. doi: 10.1016/j.ijpara.2016.09.005 (PMC5297353; doi:10.1016/j.ijpara.2016.09.005)
Supplement: Supplementary Figs. S1–S5 [file mmc1.docx]

**Supplementary Fig. S1**

**Supplementary Fig. S2**

**A**

**B**

**Supplementary Fig. S3**

**B**

**A**

**Supplementary Fig. S4**

**A**

**B**

**Supplementary Fig. S5**

**A**

**B**
