# Supplementary material for: Cord blood IgG and the risk of severe Plasmodium falciparum malaria in the first year of life
Source: Int J Parasitol. 2017 Feb;47(2-3):153–62. doi: 10.1016/j.ijpara.2016.09.005 (PMC5297353; doi:10.1016/j.ijpara.2016.09.005)
Supplement: Supplementary Table S1 [file mmc2.docx]

| Antigen | Rate of decay^a^ (95% CI)  Cases  (*n*=20) | Rate of decay^b^ (95% CI)  Controls  (*n*=54) |
| --- | --- | --- |
| AMA1 (3D7 *P. falciparum* strain) | -0.20(-0.25, -0.15) | -0.23(-0.26, -0.18) |
| MSP-2 (Dd2 *P. falciparum* strain) | -0.20(-0.22, -0.10) | -0.26(-0.22, -0.28) |
| MSP-3 (3D7) | -0.23(-0.31, -0.17) | -0.27(-0.35, -0.21) |
| MSP-1_19_ | -0.13(-0.17, -0.09) | -0.17(-0.22, -0.12) |
| *Pf*Rh2 | -0.11(-0.14, -0.01) | -0.10(-0.12, -0.05) |

**Supplementary Table S1.** A comparison of the rate of decay of specific merozoite antigens among severe malaria cases and controls in this study.

^a^Decay rate in cases (Log_10_AU per month).

^b^ Decay rate in controls (Log_10_AU per month).

CI, Confidence interval; AMA, apical membrane antigen; MSP, merozoite surface protein; *Pf*Rh, *Plasmodium falciparum* reticulocyte-binding homolog; AU, arbitrary units
